# Sonochemical‐Assisted Synthesis of Ultrathin NiCu layered Double Hydroxide for Enhanced C—N Coupling toward Electrocatalytic Urea Synthesis

**DOI:** 10.1002/smsc.202300150

**Published:** 2023-12-10

**Authors:** Hele Guo, Siyu Fu, Guohao Xue, Feili Lai, Tianxi Liu

**Affiliations:** ^1^ The Key Laboratory of Synthetic and Biological Colloids Ministry of Education, School of Chemical and Material Engineering International Joint Research Laboratory for Nano Energy Composites Jiangnan University Wuxi 214122 P. R. China; ^2^ Department of Chemistry KU Leuven Celestijnenlaan 200F 3001 Leuven Belgium

**Keywords:** C—N coupling, layered double hydroxides, ultrasonic-assisted solvothermal synthesis, urea synthesis

## Abstract

The electrocatalytic carbon–nitrogen (C—N) coupling, facilitating one‐step urea synthesis under ambient conditions, holds great promise as a viable alternative to conventional protocols. However, developing efficient and low‐cost electrocatalysts for C—N coupling remains a great challenge. Herein, a “bottom‐up” strategy is proposed to synthesize multidimensional hybrid materials of ultrathin NiCu layered double hydroxide (LDH) nanosheets on carbon nanofiber (u‐NiCu‐LDH/CNF) through an ultrasonic‐assisted solvothermal method. The NiCu‐LDH nanosheets in the u‐NiCu‐LDH/CNF composite exhibit a significantly thinner morphology compared to NiCu‐LDH/CNF prepared by conventional solvothermal without ultrasonic assistance. Leveraging its large specific surface area and well‐exposed active sites, the u‐NiCu‐LDH/CNF demonstrates dramatically improved electrocatalytic activity in C—N coupling for urea production, leading to a satisfactory urea yield rate (19.43 mmol g^−1^ h^−1^) and a high Faradaic efficiency (13.95%). Density functional theory calculations reveal that the C—N coupling step on the NiCu‐LDH model starts through the reaction between *NO_2_ and *CO_2_ intermediates. This spontaneous C—N coupling process is beneficial in promoting high levels of urea yield. This work presents a facile approach for preparing 2D ultrathin LDH, showcasing tremendous prospects in electrocatalytic urea synthesis.

## Graphical abstract

A multidimensional hybrid material of ultrathin NiCu layered double hydroxide (LDH) nanosheets (<2 nm) on carbon nanofiber (u‐NiCu‐LDH/CNF) is synthesized through an ultrasonic‐assisted solvothermal method, which shows stable and significantly enhanced electrocatalytic activity in C—N coupling of urea production due to its robust structure, large specific surface area, and fully exposed active sites.

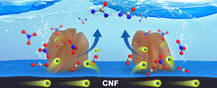

## 1 Introduction

Urea, known for its high nitrogen content, serves as a vital fertilizer in agriculture and a key raw material in the chemical industry.^[^ ^1^, ^2^ ^]^ Conventional urea production involves two sequential processes: the conversion of N_2_ and H_2_ to ammonia (NH_3_) (N_2_ + 3H_2_ → 2NH_3_), followed by the reaction between NH_3_ and CO_2_ to form urea (2NH_3_ + CO_2_ → CO(NH_2_)_2_ + H_2_O).^[^ ^3^, ^4^ ^]^ However, the widely used Haber–Bosch process for N_2_ conversion to NH_3_ requires harsh conditions, including high temperatures (400–500 °C) and pressures (100–200 bar), resulting in significant energy consumption and greenhouse gas emissions.^[^ ^5^, ^6^, ^7^, ^8^ ^]^ Hence, it is crucial and urgent to develop an efficient and environmentally friendly urea synthesis system. The electrochemical synthesis of urea, driven by renewable electricity under mild temperatures and pressures, represents a highly promising approach, which not only offers economic feasibility and environmental benefits but also enables efficient and sustainable urea production.^[^ ^9^ ^]^ Considering the low dissociation energy of N=O bond (204 kJ mol^−1^) and the high solubility of NO_3_^−^ in aqueous electrolytes, electrocatalytic C—N coupling through NO_3_^−^ and CO_2_ electrolysis emerges as a superior and promising alternative for direct urea synthesis, as compared to the utilization of N_2_ for urea production.^[^ ^10^, ^11^, ^12^, ^13^ ^]^ Despite the demonstrated improvements in Faradaic efficiency (FE) and yield rate achieved by noble metal‐based catalysts and single‐atom electrocatalysts for electrocatalytic urea synthesis,^[^ ^14^, ^15^, ^16^ ^]^ their high cost and complex preparation processes hinder their widespread application. Therefore, a significant challenge lies in the development of cost‐effective electrocatalysts with high catalytic activity and stability for urea synthesis, aiming to contribute to the economic feasibility of large‐scale urea production.

Layered double hydroxides (LDHs) are typical 2D materials consisting of positively charged metal hydroxide layers intercalated with anions and water molecules.^[^ ^17^, ^18^, ^19^ ^]^ Benefiting from their unique layered structure, tunable composition, and easy large‐scale preparation, they have become promising candidates in electrocatalytic applications, including water splitting, urea oxidation reaction, methanol oxidation reaction, and so on.^[^ ^20^, ^21^, ^22^ ^]^ However, the application of LDH in the field of electrocatalytic synthesis of urea is still blank. To date, there are some reported research works showing that Cu‐ and Ni‐based materials can facilitate the adsorption of CO_2_ and NO_3_^−^, thus being effective catalysts for the C—N coupling process.^[^ ^15^, ^23^, ^24^ ^]^ Therefore, the development of NiCu‐LDH may achieve unexpected C—N coupling activity. To fully unleash their potential, the layered materials usually require exfoliation processes.^[^ ^25^ ^]^ Compared with bulk LDH, exfoliated ultrathin LDH nanosheets exhibit increased surface area and higher density of catalytically active sites with exposed edge and planar atoms.^[^ ^26^, ^27^ ^]^ Currently, several methods have been systematically developed for the preparation of ultrathin LDHs by liquid exfoliation method.^[^ ^28^, ^29^, ^30^ ^]^ However, traditional “top‐down” liquid exfoliation methods are typically characterized by their high cost, time‐consuming nature, and the requirement of toxic compounds.^[^ ^31^, ^32^ ^]^ In addition, the exfoliated LDH nanosheets tend to be significantly occupied by adsorbed solvent molecules, hindering the surface‐active sites and reducing the catalytic activity.^[^ ^31^, ^33^ ^]^ Once the solvent is removed to obtain a powdered product, LDH nanosheets have a tendency to reaggregate into bulk LDH.^[^ ^26^ ^]^ Developing effective approaches for the preparation of ultrathin and stable NiCu‐LDH nanosheets holds immense importance in advancing the realm of electrocatalysis, particularly for the challenging task of electrocatalytic C—N coupling.

In this study, we present a facile “bottom‐up” approach to synthesize ultrathin NiCu‐LDH nanosheets (<2 nm) on carbon nanofiber (CNF) substrates (u‐NiCu‐LDH/CNF) using an ultrasonic‐assisted solvothermal method. By incorporating ultrasonic assistance during synthesis, the resulting NiCu‐LDH nanosheets in u‐NiCu‐LDH/CNF composite demonstrate a remarkably thinner average thickness and a larger electrochemical surface area compared to NiCu‐LDH/CNF prepared without ultrasonic assistance. These structural advantages contribute to the significantly enhanced electrocatalytic activity and stability of u‐NiCu‐LDH/CNF for the carbon–nitrogen (C—N) coupling reaction in urea production. Specifically, the FE and urea yield rate of the u‐NiCu‐LDH/CNF composite are 2.7 and 1.5 times those of NiCu‐LDH/CNF, respectively. The findings from our research provide a novel and innovative approach for the synthesis of ultrathin 2D materials tailored for efficient electrocatalytic urea synthesis.

## 2 Result and Discussion

**Figure 1:**
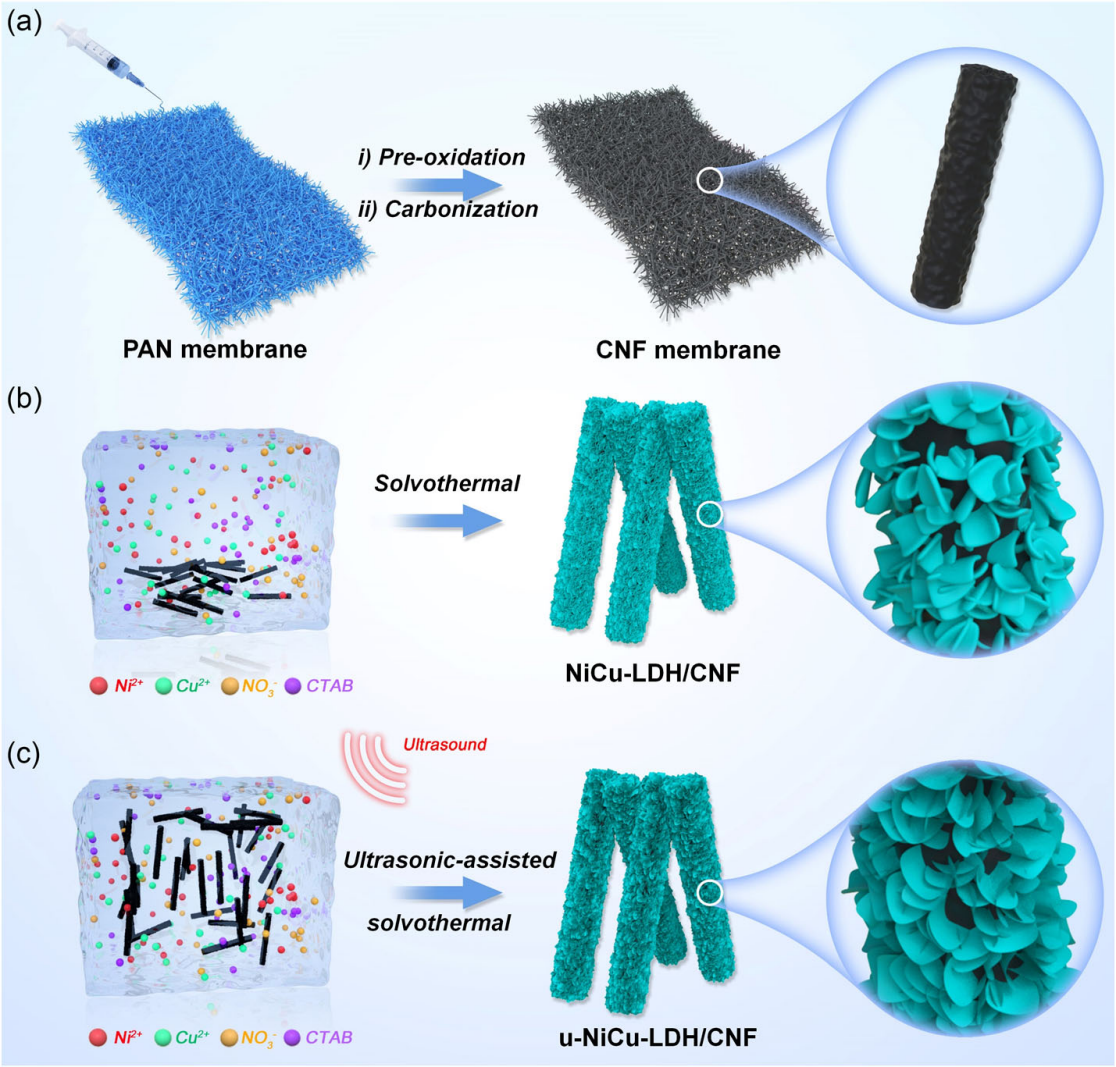
a–c) Schematic illustrations of: a) the preparation of CNF substrates by electrospinning PAN membrane and followed by peroxidation and carbonization process; b) the preparation of NiCu‐LDH/CNF by the conventional solvothermal method; and c) the preparation of u‐NiCu‐LDH/CNF by the ultrasound‐assisted solvothermal method.

**Figure 2:**
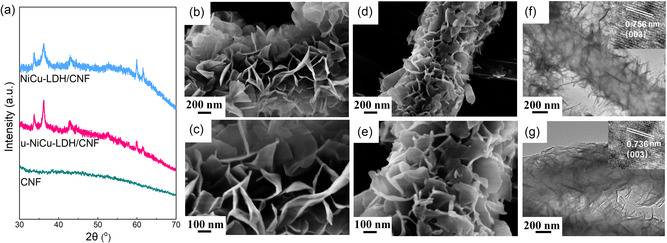
a) XRD patterns of CNF, u‐NiCu‐LDH/CNF, and NiCu‐LDH/CNF composites. b–e) SEM images of u‐NiCu‐LDH/CNF (b,c) and NiCu‐LDH/CNF (d,e) composites. f,g) TEM images of u‐NiCu‐LDH/CNF (f) and NiCu‐LDH/CNF (g) composites. The insets of (f,g) are the HRTEM images of u‐NiCu‐LDH/CNF and NiCu‐LDH/CNF, respectively.

The synthesis processes of u‐NiCu‐LDH/CNF and NiCu‐LDH/CNF composites are illustrated in **Figure** **1**. First, the electrospun polyacrylonitrile (PAN) membrane was subjected to preoxidation, followed by carbonization under N_2_ flow to produce CNFs, as shown in Figure 1a. The NiCu‐LDH/CNF composite was then synthesized through a conventional solvothermal method using a solution containing pulverized CNFs and metal ions of Ni^2+^ and Cu^2+^ (Figure 1b), while the u‐NiCu‐LDH/CNF composite was obtained via an ultrasonic‐assisted solvothermal process (Figure 1c). The X‐ray diffraction (XRD) patterns of CNF, u‐NiCu‐LDH/CNF, and NiCu‐LDH/CNF composites are shown in **Figure** **2**a. The characteristic peaks at around 33.7°, 36.2°, 42.9°, 59.9°, and 61.6° can be observed in u‐NiCu‐LDH/CNF and NiCu‐LDH/CNF composites, and correspond to the typical crystal planes of α‐Ni(OH)_2_ (JCPDS#‐38‐0715). Note that the introduction of Cu into Ni(OH)_2_ may cause structural transformation, causing some shifts of the patterns.^[^ ^34^ ^]^ Field‐emission scanning electron microscopy (FESEM) was performed to observe the morphologies of u‐NiCu‐LDH/CNF and NiCu‐LDH/CNF composites. Figure 2b,c depict the FESEM images of the u‐NiCu‐LDH/CNF composite, revealing a plethora of smooth and flexible nanosheets that anchor on the CNF substrates uniformly and vertically. In contrast, the NiCu‐LDH nanosheets with smaller sizes grow on the CNF substrate disorderly to generate the NiCu‐LDH/CNF composite (Figure 2d,e). Besides, energy‐dispersive X‐ray spectroscopy reveals the presence of Ni, Cu, O, and C elements in u‐NiFe‐LDH/CNF (Figure S1, Supporting Information). Transmission electron microscopy (TEM) image of the u‐NiCu‐LDH/CNF composite further reveals that the surface of CNFs with a diameter of around 400 nm is covered by abundant ultrathin LDH nanosheets vertically and uniformly (Figure 2f). For comparison, the NiCu‐LDH/CNF composite contains relatively disordered and obliquely grown LDH nanosheets (Figure 2g). This result suggests that ultrasound can promote the ordered and vertical growth of large‐sized NiCu‐LDH nanosheets during solvothermal synthesis, which is beneficial for the sufficient exposure of active sites and the rapid transport of ions during the subsequent electrocatalytic reaction. In addition, the high‐resolution TEM (HRTEM) image confirms that the lattice fringe spacing of the (003) crystal plane of NiCu‐LDH in the u‐NiCu‐LDH/CNF composite is 0.756 nm (inset of Figure 2f), which is slightly larger than that of the NiCu‐LDH/CNF composite (0.736 nm, inset of Figure 2g). This is mainly attributed to the stripping effect of ultrasonic waves on NiCu‐LDH nanosheets. High‐angle annular dark‐field scanning TEM images and corresponding elemental mappings demonstrate the coexistence of Ni, Cu, and O elements throughout the NiCu‐LDH nanosheets of u‐NiCu‐LDH/CNF and NiCu‐LDH/CNF composites (Figure S2 and S3, Supporting Information). To better show the different metal contents in u‐NiCu‐LDH/CNF and NiCu‐LDH/CNF composites, the inductively coupled plasma mass spectrometry was carried out with detailed information in Table S1, Supporting Information. The results indicate that the introduction of ultrasound during the solvothermal process will not affect the elemental composition of the as‐prepared NiCu‐LDH nanosheets.

**Figure 3:**
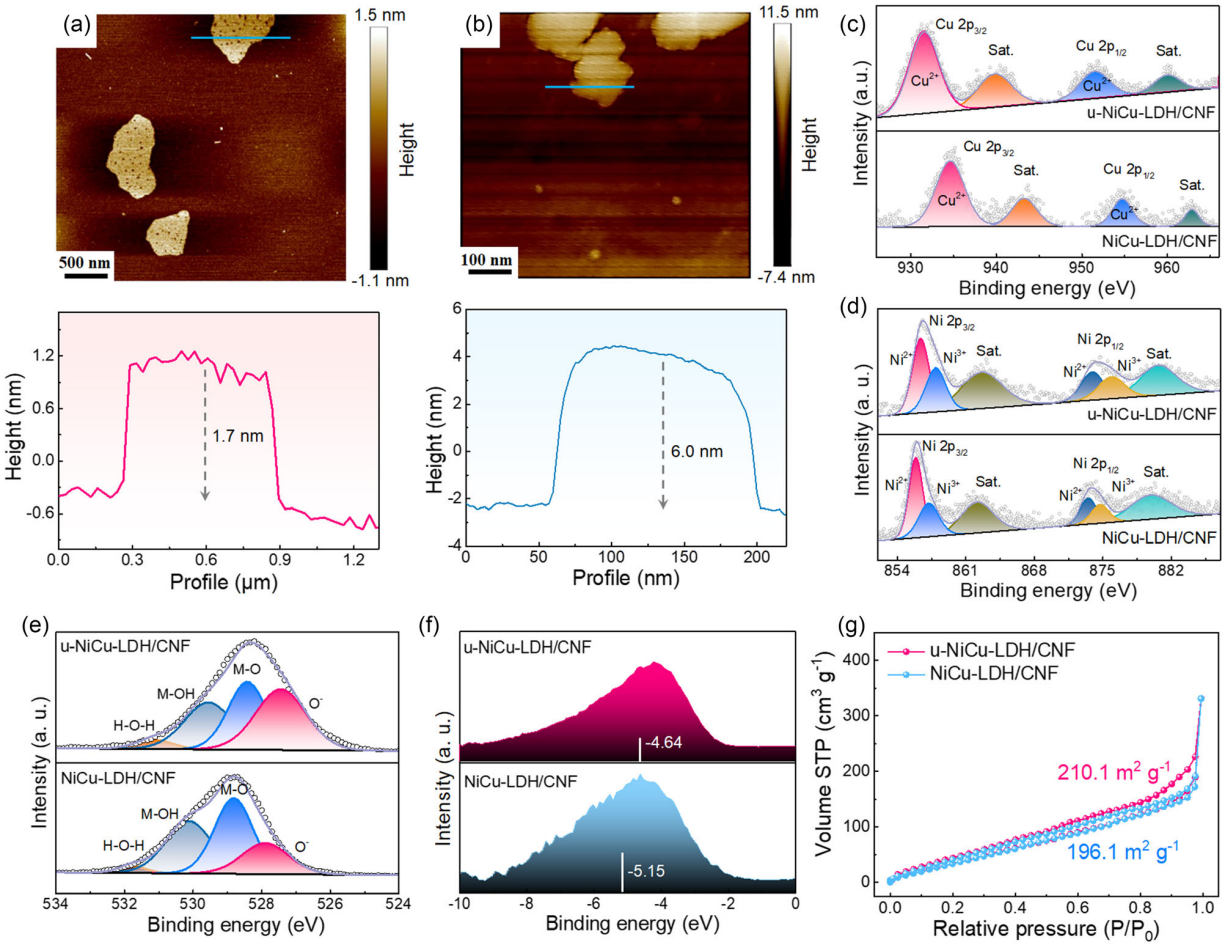
a,b) AFM images of u‐NiCu‐LDH/CNF (a) and NiCu‐LDH/CNF (b) composites. c–e) High‐resolution Cu 2p (c), Ni 2p (d), and O 1s (e) spectra of u‐NiCu‐LDH/CNF and NiCu‐LDH/CNF composites. f) The valence‐band XPS spectra of u‐NiCu‐LDH/CNF and NiCu‐LDH/CNF composites. g) Nitrogen sorption‐desorption isotherms of u‐NiCu‐LDH/CNF and NiCu‐LDH/CNF composites.

The thicknesses of NiCu‐LDH nanosheets in both u‐NiCu‐LDH/CNF and NiCu‐LDH/CNF composites were examined using atomic force microscopy (AFM). In **Figure** **3**a, the average thickness of the NiCu‐LDH nanosheets for the u‐NiCu‐LDH/CNF composite was measured to be about 1.7 nm, which is significantly thinner compared to the NiCu‐LDH nanosheets (6.0 nm) in the NiCu‐LDH/CNF composite (Figure 3b). The introduction of ultrasound in the solvothermal process can promote the formation of more uniform and thinner NiCu‐LDH nanosheets, which mainly originates from the following reasons. First, the application of ultrasonic waves during the synthesis process enhances the dispersion and agitation of the reaction mixture.^[^ ^35^ ^]^ This promotes a relatively uniform distribution of precursors and facilitates the nucleation and growth of NiCu‐LDH nanosheets on CNF substrates. Second, the ultrasonic waves assist in breaking down large agglomerates or particles, which can result in a homogeneous precursor dispersion.^[^ ^36^ ^]^ This improved dispersion is beneficial to enabling enhanced assembly of metal cations and anions, thus facilitating the formation of ultrathin NiCu‐LDH nanosheets. Furthermore, the application of ultrasonic waves can enhance mass transfer and diffusion processes during the solvothermal reaction.^[^ ^37^ ^]^ It allows for efficient and rapid precursor conversion, reducing the reaction time, and promoting the formation of ultrathin NiCu‐LDH nanosheets.

X‐ray photoelectron spectroscopy (XPS) was carried out to elucidate the chemical compositions and states of u‐NiCu‐LDH/CNF and NiCu‐LDH/CNF composites. As shown in Figure S4, Supporting Information, C, O, Ni, and Cu elements are clearly presented on the full survey spectra of u‐NiCu‐LDH/CNF and NiCu‐LDH/CNF composites. High‐resolution Cu 2p spectra of u‐NiCu‐LDH/CNF and NiCu‐LDH/CNF are displayed in Figure 3c. High‐resolution Cu 2p spectrum of NiCu‐LDH/CNF can be deconvoluted into four peaks, which are related to the Cu^2+^ 2p_3/2_ (934.58 eV), Cu^2+^ 2p_1/2_ (954.73 eV), and two satellite peaks (943.38 and 962.76 eV), respectively.^[^ ^38^, ^39^ ^]^ However, the binding energies of Cu^2+^ 2p_3/2_ and Cu^2+^ 2p_1/2_ signals in u‐NiCu‐LDH/CNF are negatively shifted compared with those in NiCu‐LDH/CNF, indicating a decreased oxidation state of the Cu species.^[^ ^40^ ^]^ High‐resolution Ni 2p spectrum of NiCu‐LDH/CNF can be divided into six distinct peaks (Figure 3d), belonging to Ni^2+^ (855.80 and 873.40 eV), Ni^3+^ (857.09 and 874.57 eV) species, and two satellite peaks (862.25 and 879.96 eV).^[^ ^41^, ^42^ ^]^ A positive shift of the binding energy for Ni^2+^ and Ni^3+^ signals can be observed for u‐NiCu‐LDH/CNF by comparison with those of NiCu‐LDH/CNF, suggesting an increased oxidation state of the Ni species (increased content of Ni^3+^) under ultrasound.^[^ ^43^ ^]^ High‐resolution O 1*s* spectra indicate that the presence of surface labile oxygen (O^−^), lattice oxygen (M–O), metal hydroxide (M–OH), and adsorbed water molecular (H–O–H) in both u‐NiCu‐LDH/CNF and NiCu‐LDH/CNF composites (Figure 3e).^[^ ^44^, ^45^ ^]^ Additionally, the valence‐band XPS technique was employed to assess the d‐band centers of u‐NiCu‐LDH/CNF and NiCu‐LDH/CNF composites. As shown in Figure 3f, the d‐band center of the u‐NiCu‐LDH/CNF composite (−4.64 eV) exhibits an upward shift in comparison to that of the NiCu‐LDH/CNF composite (−5.15 eV), suggesting that u‐NiCu‐LDH/CNF possesses a higher affinity for the reactive species (such as CO_2_ or NO_3_^−^ ions) during the electrocatalytic synthesis of urea.^[^ ^46^ ^]^ This enhanced affinity indicates the potential superiority of u‐NiCu‐LDH/CNF as an electrocatalyst for urea production. The N_2_ adsorption–desorption isotherms of u‐NiCu‐LDH/CNF and NiCu‐LDH/CNF composites were recorded to evaluate their specific surface areas (Figure 3g). The specific surface area of the u‐NiCu‐LDH/CNF composite is calculated to be 210.1 m^2^ g^−1^, which is larger than that of NiCu‐LDH/CNF (196.1 m^2^ g^−1^). The increase in specific surface area is mainly attributed to the ultrathin NiCu‐LDH nanosheets formed by the ultrasonic‐assisted solvothermal process.^[^ ^47^ ^]^

**Figure 4:**
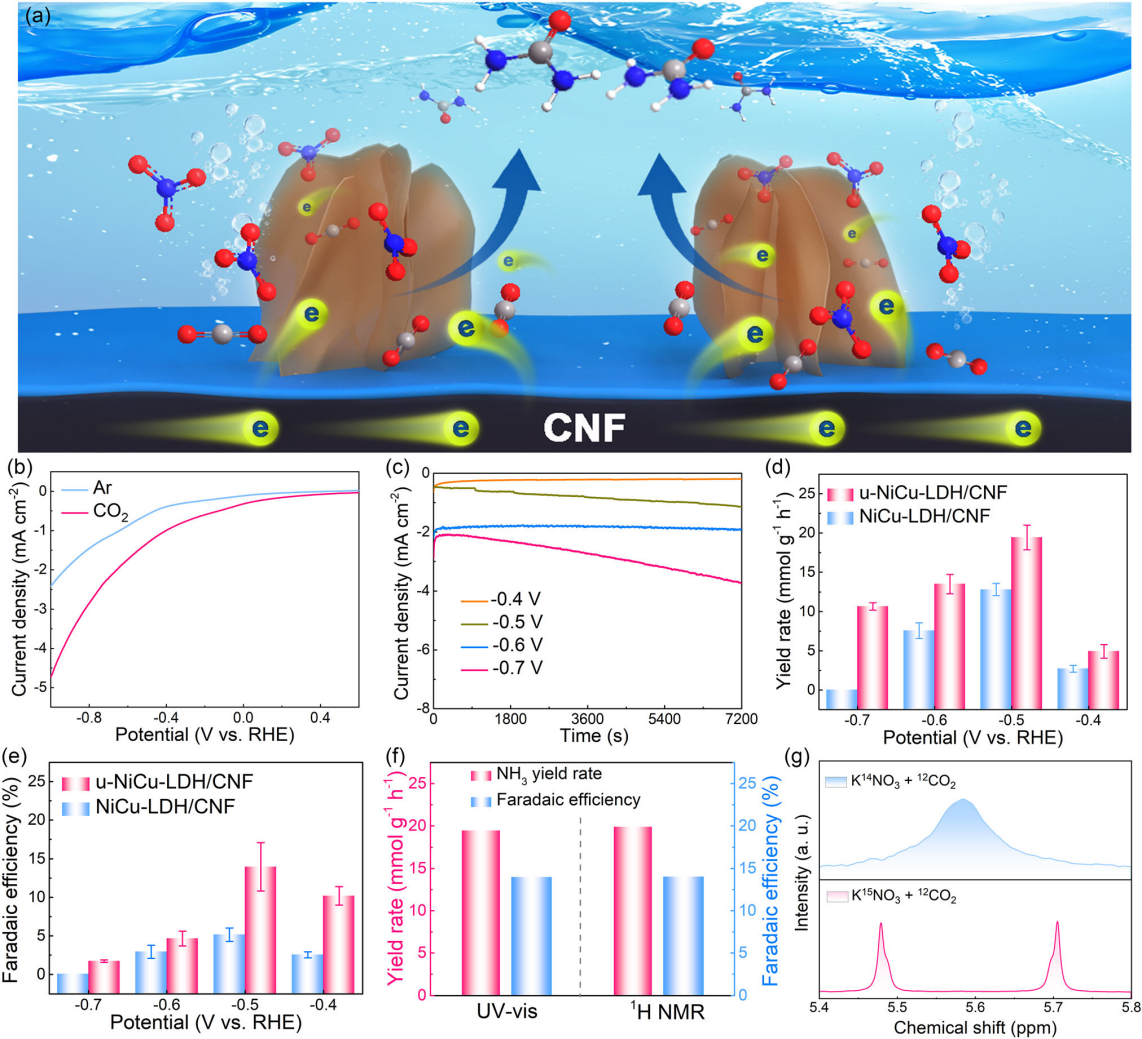
a) Schematic illustration of the u‐NiCu‐LDH/CNF composite for electrocatalytic synthesis of urea. b) LSV curves for u‐NiCu‐LDH/CNF composite in Ar‐ and CO_2_‐saturated 0.1 m KNO_3_ electrolytes. c) Chronoamperometry results of u‐NiCu‐LDH/CNF composite at different potentials. d) Urea yield rate and e) FE of u‐NiCu‐LDH/CNF and NiCu‐LDH/CNF composites under different potentials. f) Urea yield rate and FE of u‐NiCu‐LDH/CNF composite after 2 h of electrolysis (at −0.5 V vs RHE) determined using the UV–vis and ^1^H NMR spectra. g) ^1^H NMR spectra of urea prepared with K^15^NO_3_ and K^14^NO_3_ as nitrogen sources, respectively.

The electrochemical syntheses of urea by u‐NiCu‐LDH/CNF and NiCu‐LDH/CNF composites were performed in an H‐type electrolyzer with a CO_2_‐saturated 0.1 m KNO_3_ electrolyte. **Figure** **4**a shows the electrocatalytic C—N coupling process with NO_3_^−^ and CO_2_ as reactants on the u‐NiCu‐LDH/CNF surface. Figure 4b and Figure S5, Supporting Information, show linear sweep voltammetry (LSV) curves of u‐NiCu‐LDH/CNF and NiCu‐LDH/CNF composites in CO_2_/Ar‐saturated 0.1 m KNO_3_ electrolyte. An enhanced current density can be observed in the CO_2_‐saturated electrolyte for both u‐NiCu‐LDH/CNF and NiCu‐LDH/CNF composites, indicating the occurrences of the electrocatalytic urea synthesis process. The electrocatalytic activities of u‐NiCu‐LDH/CNF and NiCu‐LDH/CNF composites for urea synthesis were evaluated by calculating the yield rate of urea after chronoamperometry test at different potentials ranging from −0.4 to −0.7 V vs reversible hydrogen electrode (RHE) for 2 h (Figure 4c and S6, Supporting Information). The yield of urea was quantitatively determined by the diacetyl monoxime method.^[^ ^48^ ^]^ The u‐NiCu‐LDH/CNF composite achieves a satisfactory maximum urea yield rate of 19.43 mmol g^−1^ h^−1^ and the highest FE value of 13.95% at −0.5 V vs RHE, which are much better than those of NiCu‐LDH/CNF composite (12.80 mmol g^−1^ h^−1^ and 5.15%), as shown in Figure 4d,e. For comparison, the performance of u‐NiCu‐LDH and NiCu‐LDH are displayed in Figure S7, Supporting Information. Both NiCu‐LDH/CNF and u‐NiCu‐LDH/CNF composites exhibit higher urea yield rates and FEs than their counterparts lacking CNFs as conductive supports (Figure 4d,e and Figure S7, Supporting Information), suggesting that the conductive CNF can promote the electrocatalytic urea synthesis significantly. Figure S8, Supporting Information shows the product distributions during the urea synthesis at different potentials on the u‐NiCu‐LDH/CNF composite. Four main products of H_2_, CO, NH_3_, and NH_2_CONH_2_ can be detected. According to the product distribution in Figure S8, Supporting Information, the Ni and Cu diatomic sites in NiCu‐LDH nanosheets may trigger a large number of activated C and N species simultaneously, and provide the possibility for a chemical coupling process to generate C—N bonds. The by‐product of NH_3_ during electrocatalytic urea synthesis is detectable, which may affect the accuracy of urea determination using the diacetyl monoxime method.^[^ ^49^, ^50^ ^]^ Therefore, the ^1^H NMR method was also employed to measure the produced urea quantitatively and verify the reliability of the diacetyl monoxime method simultaneously (Figure S9, Supporting Information). The results demonstrate that the urea yield rate measured by the ^1^H NMR method is essentially consistent with that detected by the colorimetric method, confirming the accuracies of the used quantitative methods (Figure 4f). Table S2, Supporting Information includes the catalytic activity, FE, and stability of recently reported electrocatalysts for urea production, underscoring the significant potential of NiCu‐LDH/CNF as a promising electrocatalyst for urea synthesis. To verify the nitrogen source of the synthesized urea, isotope labeling experiments were conducted using CO_2_‐saturated 0.1 m K^15^NO_3_ and K^14^NO_3_ electrolytes, respectively. The formed ^15^NH_2_CO^15^NH_2_ and ^14^NH_2_CO^14^NH_2_ products were confirmed by ^1^H NMR spectra based on the chemical shifts of the doublet and singlet couplings, respectively.^[^ ^51^ ^]^ Figure 4g illustrates the ^1^H NMR spectrum of urea obtained using CO_2_‐saturated 0.1 m K^15^NO_3_ electrolyte, which displays two characteristic peaks from ^15^NH_2_CO^15^NH_2_ product at 5.48 and 5.71 ppm. In contrast, the ^1^H NMR spectrum of the electrolyte containing K^14^NO_3_ displays only one characteristic peak from ^14^NH_2_CO^14^NH_2_ product at 5.62 ppm. This isotope labeling experiment proves that the nitrogen in the synthesized urea comes from nitrate. In addition, the ^13^C NMR spectrum of ^13^CO_2_‐saturated electrolyte after 2 h of electrocatalysis exhibits a single characteristic peak corresponding to standard ^13^C‐labeled urea (Figure S10, Supporting Information), indicating that no other carbon‐containing liquid products can be produced during the electrocatalytic urea synthesis process.^[^ ^51^ ^]^

**Figure 5:**
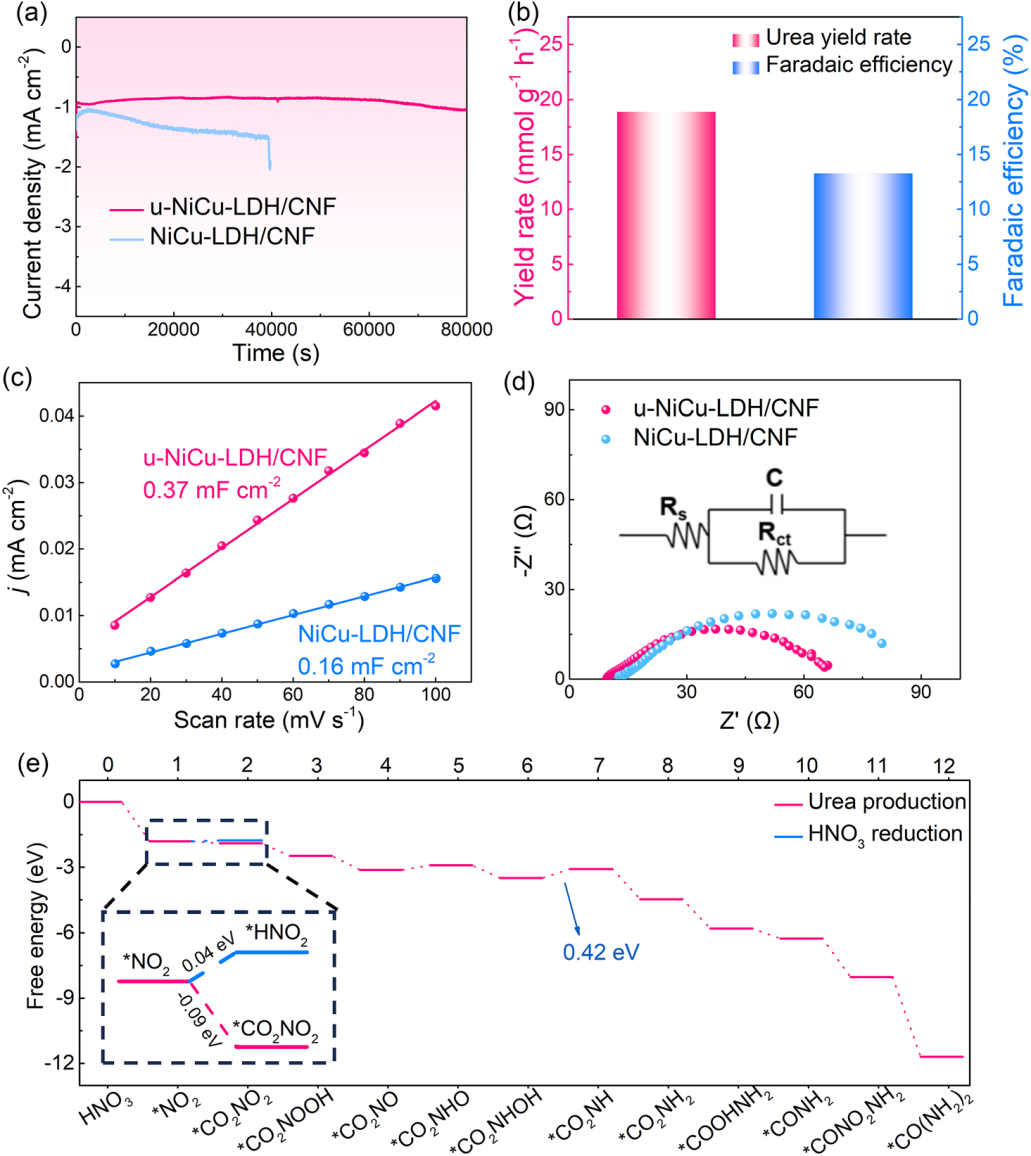
a) Long‐term stability test of NiCu‐LDH/CNF and u‐NiCu‐LDH/CNF composites at −0.5 V vs RHE in 0.1 m KNO_3_ electrolyte saturated with CO_2_ gas. b) Urea yield rate and FE of u‐NiCu‐LDH/CNF composite after long‐term stability test. c) The *C*_dl_ values of u‐NiCu‐LDH/CNF and NiCu‐LDH/CNF composites. d) Nyquist plots of u‐NiCu‐LDH/CNF and NiCu‐LDH/CNF composites. e) Free energy diagram for urea production on the surface of NiCu‐LDH model.

The long‐term stabilities of u‐NiCu‐LDH/CNF and NiCu‐LDH/CNF composites were assessed by the chronoamperometry test (**Figure** **5**a). The current density of NiCu‐LDH/CNF exhibits significant fluctuation after testing for 11 h. However, the current density of u‐NiCu‐LDH/CNF shows remarkable stability even after more than 20 h. After the chronoamperometric response testing for more than 20 h (Figure 5b), the urea yield and FE of the u‐NiCu‐LDH/CNF composite remain almost unchanged (18.86 mmol g^−1^ h^−1^ and 13.23%), indicating its excellent catalytic stability. Figure S11, Supporting Information also demonstrates the unchanged crystal structure of LDH in the u‐NiCu‐LDH/CNF composite after long‐term stability testing. The overall activity of the electrocatalyst is influenced by the number of active sites and the intrinsic activity of each site. To characterize the electrochemical active surface areas of u‐NiCu‐LDH/CNF and NiCu‐LDH/CNF composites, capacitive currents were measured at various scan rates (ranging from 10 to 100 mV s^−1^) in non‐Faraday potential regions (Figure S12, Supporting Information). The *C*_dl_ value of the u‐NiCu‐LDH/CNF composite is calculated to be 0.37 mF cm^−2^, which is much higher than that of the NiCu‐LDH/CNF composite (0.16 mF cm^−2^). This indicates the ultrathin NiCu‐LDH nanosheets in the u‐NiCu‐LDH/CNF composite can provide more active sites during the electrocatalytic C—N coupling process. Besides, the electrochemical impedance spectroscopy analysis of u‐NiCu‐LDH/CNF and NiCu‐LDH/CNF composites are shown in Figure 5d. The u‐NiCu‐LDH/CNF composite manifests lower charge transfer resistance than NiCu‐LDH/CNF in the high‐frequency region, suggesting that the reduced thickness of NiCu‐LDH nanosheets in u‐NiCu‐LDH/CNF is beneficial to accelerating ion diffusion and reducing charge transfer resistance.

It has been reported that Ni‐based catalysts respond selectively to the adsorption and activation of CO_2_, while Cu‐based catalysts can promote the adsorption and activation of NO_3_^−^.^[^ ^15^, ^23^, ^24^ ^]^ Therefore, the synergistic effect of Ni and Cu sites in NiCu‐LDH/CNF enables efficient electrocatalytic C—N coupling. The density functional theory calculations were subsequently employed to elucidate the catalytic reaction mechanisms involved in urea synthesis. According to the free energy diagram of the energy path shown in Figure 5e, urea production starts from the thermodynamic spontaneous reduction of NO_3_^−^ (HNO_3_) on NiCu‐LDH to the *NO_2_ intermediate (−1.81 eV). Compared to the 0.04 eV energy barrier observed for the protonation of *NO_2_ to form *HNO_2_, the formation of *CO_2_NO_2_ requires only a lower energy barrier of −0.09 eV (inset of Figure 5e). This low energy barrier favoring *CO_2_NO_2_ formation plays a beneficial role in promoting the direct C—N coupling process in the early stages. The spontaneous progression of the formation of *NO_2_ and the C—N coupling process may synergistically contribute to the high levels of urea yield rate. Subsequent protonation processes are more likely to take place on the *NO_2_ moiety of *CO_2_NO_2_, leading to the formation of the *CO_2_NH_2_ intermediate. During this protonation process, the free energy required for the formation of *CO_2_NH intermediate from CO_2_NHOH is 0.42 eV, which is the rate‐determining step in the overall urea production process. Then, the formation of urea follows a thermodynamically spontaneous pathway. The *CONH_2_ intermediate is formed first, and the *CONO_2_NH_2_ intermediate is produced by coupling the second *NO_2_. Finally, *CONO_2_NH_2_ is further protonated to form *CO(NH_2_)_2_.

## 3 Conclusion

A “bottom‐up” strategy is proposed to assemble ultrathin NiCu‐LDH nanosheets on PAN‐derived CNF through an ultrasonic‐assisted solvothermal method. Under the influence of ultrasonic waves, the anchored NiCu‐LDH nanosheet has a thickness of 1.7 nm, which is considerably thinner than that of NiCu‐LDH nanosheet prepared without ultrasonic assistance. Benefiting from its robust multidimensional hybrid structure, large specific surface area, and well‐exposed active sites, u‐NiCu‐LDH/CNF exhibits stable and significantly enhanced electrocatalytic activity in C—N coupling for urea production, including a satisfactory urea yield of 19.43 mmol g^−1^ h^−1^ and a high FE of 13.95%. This research introduces a straightforward and highly efficient approach for engineering ultrathin 2D materials, which not only addresses the limitations associated with conventional liquid‐phase exfoliation techniques, but also demonstrates substantial promise in the realm of advanced catalytic fields, particularly in the context of synergistic catalytic C—N coupling.

## Conflict of Interest

The authors declare no conflict of interest.

## Supporting information

Supplementary Material

Supplementary Material

## Data Availability

The data that support the findings of this study are available from the corresponding author upon reasonable request.
