# Supplementary material for: Sonochemical‐Assisted Synthesis of Ultrathin NiCu layered Double Hydroxide for Enhanced C—N Coupling toward Electrocatalytic Urea Synthesis
Source: Small Sci. 2023 Dec 10;5(7):2300150. doi: 10.1002/smsc.202300150 (PMC12257894; doi:10.1002/smsc.202300150)
Supplement: Supplementary file 1 — Supplementary Material [file SMSC-5-2300150-s002.pdf]

Supporting Information

Sonochemical-Assisted Synthesis of Ultrathin NiCu Layered Double Hydroxide for Enhanced C−N Coupling toward Electrocatalytic Urea Synthesis

Hele Guo[a#], Siyu Fu[a#], Guohao Xue[a], Feili Lai[b*] and Tianxi Liu[a*]

[a] The Key Laboratory of Synthetic and Biological Colloids, Ministry of Education, School of Chemical and Material Engineering, International Joint Research Laboratory for Nano Energy Composites, Jiangnan University, Wuxi 214122, P. R. China

[b] Department of Chemistry, KU Leuven, Celestijnenlaan 200F, Leuven 3001, Belgium

[*] Corresponding authors (emails: feili.lai@kuleuven.be; txliu@jiangnan.edu.cn)

Experimental section

Materials synthesis

Synthesis of electrospun CNF powder: Polyacrylonitrile (PAN) was dissolved in DMF under magnetic stirring at room temperature for 12 h to prepare a 10 wt % spinning solution. This solution was loaded into a 10 mL syringe with a stainless-steel needle (inner diameter: 0.5 mm). A high voltage of 15 kV and a feeding rate of 0.8 mL h^{-1} were applied to the spinneret. Electrospun PAN nanofibers were collected onto the rotating aluminum collector, and placed 15 cm away from the spinneret. Subsequently, the collected PAN nanofiber membranes underwent pre-oxidation by the following program: heating up to 250 ºC with a ramp rate of 2 ºC min^{-1}, followed by holding at 250 ºC for 2 h. After pre-oxidation, the PAN nanofiber membranes were carbonized at 950 °C for 0.5 h in a N_2 flow to obtain CNF membranes, with a heating rate of 3 °C min^{-1}. Finally, the CNF membranes were crushed through a crusher, and then the CNF powder was obtained by pumping and drying.

Synthesis of u-NiCu-LDH/CNF composite: The typical procedure for preparing u-NiCu-LDH/CNF composite is shown in Figure 1c. Briefly, $Ni(NO_3)_2 \cdot 6H_2O$ (0.2 mmol) and $CuCl_2 \cdot 2H_2O$ (0.1 mmol) were dissolved in 25 mL mixture solution of methanol-water with a volume ratio of 2:1. Then, 0.40 g of cetyl-trimethylammonium bromide (CTAB) was added in an ultrasonic bath. Subsequently, 42 mg of CNF was added to the above solution. The solution was kept in a hydrothermal kettle at 180 °C for 14 h with ultrasonic treatment. Then, the samples were collected by centrifugation at 12000 rpm for 20 min, and the residuum was removed by five consecutive washing/centrifugation cycles with ethanol and water. The obtained sample was denoted as u-NiCu-LDH/CNF. For comparison, the NiCu-LDH/CNF composite was prepared by the same procedure as the u-NiCu-LDH/CNF except for ultrasonic treatment.

Materials characterization

X-ray diffraction (XRD) patterns of samples were collected from a MiniFlex600 X-ray diffractometer with Cu Kα radiation (λ = 0.1542 nm) under a voltage of 40 kV and a current of 40 mA. X-ray photoelectron spectroscopy (XPS) measurements and the valence band were performed on an Axis Supra by Kratos Analytical Inc. The carbon peak at 284.8 eV was used as a reference to correct the charging effects. Scanning

electron microscopy (SEM) images were taken with a Hitachi S-4800 scanning electron microscope. Energy-dispersive X-ray spectroscopies (SEM-EDS) were taken with a Hitachi S-4800 scanning electron microscope. Transmission electron microscopy (TEM) and the high-resolution TEM (HRTEM) images were collected from a JEM-2100 plus transmission electron microscope with applying an acceleration voltage of 200 kV.

Electrochemical measurements

The electrochemical activity of u-NiCu-LDH/CNF for urea production was evaluated using an H-type electrolyzer and a CHI 660D electrochemical workstation in a standard three-electrode system. The pretreated Nafion 117 membrane (Dupont) was used as the separator, and 0.1 M KNO_3 electrolyte was used in this experiment. Before tests, the Nafion 117 membrane was pretreated by heating it in H_2O_2 (5%) aqueous solution at 80 °C for 1 h and ultrapure water at 80 °C for another 1 h, respectively, followed by treatment in 0.05 M H_2SO_4 for 1 h and ultrapure water for another 3 h. 2 mg of catalyst and 50 μL of 5% Nafion solution were dispersed in 950 μL ethanol by sonication to generate a homogeneous ink. Then, the catalyst ink was loaded onto a piece of carbon paper and dried naturally to obtain the working electrode. The reference electrode was Ag/AgCl electrode (saturated with KCl solution), and the counter electrode was a carbon rod. Prior to the electrochemical testing, the cathode portion of the electrolyte was pre-saturated by CO_2 gas. After that, the flow rate was maintained at 15 mL min^{-1} during the catalytic process. All potentials were calibrated to a reversible hydrogen electrode (RHE) using the equation of $E_{RHE} = E_{Ag/AgCl} + 0.0591 \times pH + 0.197$.

Detection of urea production

The quantification of urea concentration is by the diacetyl monoxime method. The color reagents were prepared as follows. Solution A: 100 mL of concentrated phosphoric acid was mixed with 300 mL of concentrated sulfuric acid and 600 mL of distilled water. Then, 100 mg of ferric chloride was dissolved in the above solution to obtain solution A. Solution B: 100 mg of thiosemicarbazide (TSC) and 5 g of diacetylmonoxime (DAMO) were dissolved in 1000 mL of distilled water to obtain solution B. For the color generation, 2 mL of solution A and 1 mL of solution B were added to 1 mL of urea-contained solution and mixed vigorously. Then, the solution was heated to 100 °C and maintained for 15 min. After cooling to 25 °C, the absorbance was acquired at 525

nm.

Detection of ammonia production

The quantification of ammonia concentration is by the indophenol blue method. A series of the color regents (Color regent A: 1 M NaOH solution containing 5 wt% salicylic acid and 5 wt% sodium citrate; Color regent B: 0.05 M NaClO; Color regent C: 1 g of sodium nitroferricyanide was dissolved in 100 g of water) were prepared. Then, 2 mL of electrolyte after electrocatalysis was transferred to a glass bottle. And 2 mL of color regent A, 1 mL of color regent B, and 0.2 mL of color regent C were added in turn to the above glass bottle. The absorbance was measured in the range from 500 to 800 nm after the solution was reserved in dark for 2 h.

Detection of CO and H_2

The quantitative analyses of H_2 and CO were carried out by gas chromatography (Agilent 7890a) with a thermal conductivity detector.

^{15}N isotope labeling experiment

The ^{15}N isotopic labeling experiments were conducted using 0.1 M $K^{15}NO_3$ electrolyte with CO_2 as feeding gas. After electrolysis for 2 h at -0.5 V vs. RHE, 375 μL of electrolyte solution and 125 μL of DMSO-d6 were transferred into NMR tubes for ^{1}H NMR measurements. The ^{13}C isotopic experiments were performed in 0.1 M KNO_3 electrolyte with $^{13}CO_2$ as feeding gas. After the electrolysis at -0.5 V vs. RHE for 2 h, the electrolyte was concentrated by vacuum drying at 60 °C. 375 μL of concentrated electrolyte and 125 μL of DMSO-d6 were transferred into NMR tubes for ^{13}C NMR measurements. $NH_2{}^{13}CONH_2$ was dissolved in 0.1 M KNO_3 solution (500 μL, $V_{DMSO\text{-}d6}$:V_{H2O}=1:3) as reference.

Density functional theoretical (DFT) Calculations

The DFT calculations were implemented by Vienna *ab initio* simulation package (VASP),[1-2] where the ion-electron interaction was depicted by projector augmented waves (PAW) and the exchange and correlation potential was described by the revised function of Perdew, Burke and Ernzerhof (revPBE) based on the generalized gradient

approximation (GGA).[3] In this calculation, a 3 × 7 × 1 Gamma k-points grids is used to sample the Brillouin zone for the supercell structure. Cut-off energy of 500 eV was adopted in all calculations. The vacuum space is set to be at least 20 Å to separate the interactions between the neighboring slabs. Criteria of convergence was set to 1×10^{-5} eV and 0.01 eV/Å for the self-consistent field (SCF) and ion steps, respectively.

Figures and Tables

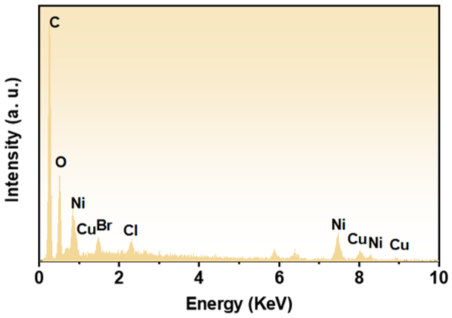

Figure S1. Energy-dispersive X-ray spectroscopy of u-NiCu-LDH/CNF composite.

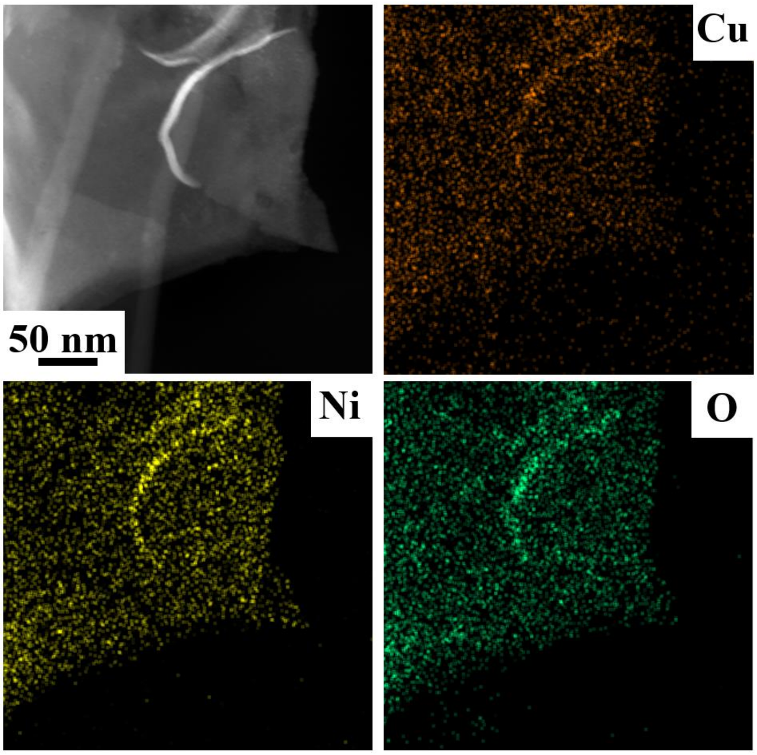

Figure S2. HAADF-STEM image and EDX elemental mappings of Cu, Ni, and O within the NiCu-LDH nanosheet of u-NiCu-LDH/CNF composite.

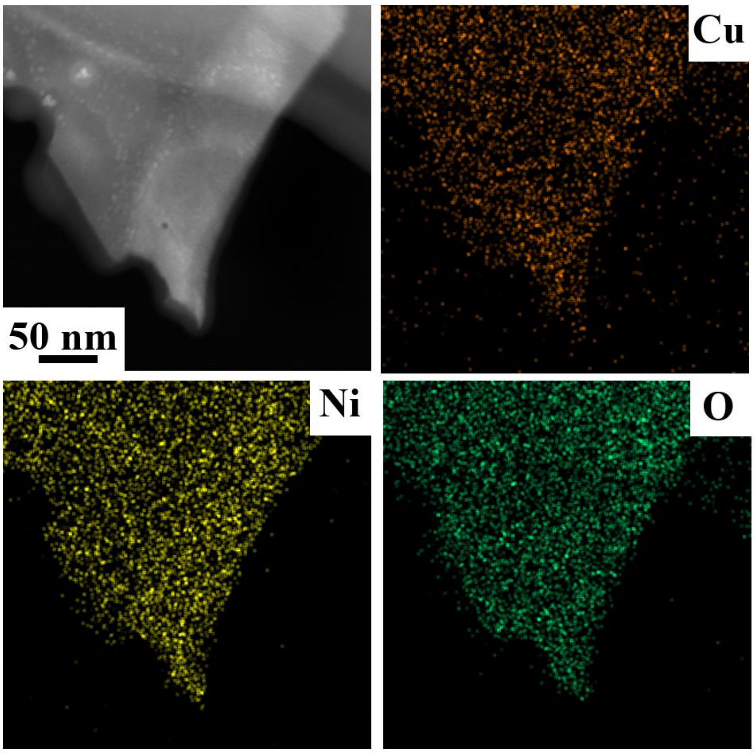

Figure S3. HAADF-STEM image and EDX elemental mappings of Cu, Ni, and O within the NiCu-LDH nanosheet of NiCu-LDH/CNF composite.

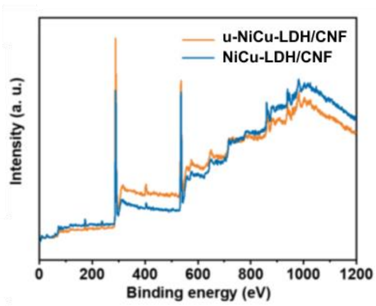

Figure S4. Full XPS curves of u-NiCu-LDH/CNF and NiCu-LDH/CNF composites.

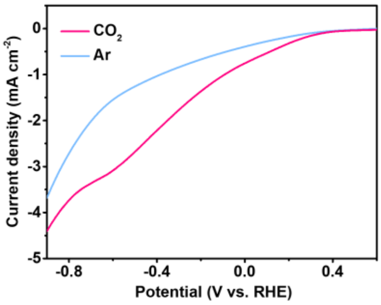

Figure S5. LSV curves of the NiCu-LDH/CNF composite in Ar- and CO_2-saturated 0.1 M KNO_3 electrolytes.

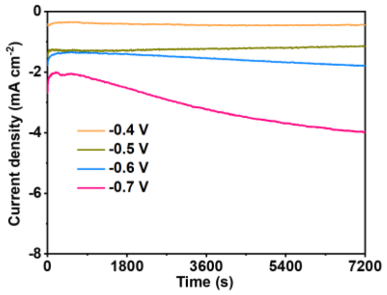

Figure S6. Chrono-amperometry results of the NiCu-LDH/CNF composite at different potentials in 0.1 M KNO_3 electrolyte with CO_2 gas.

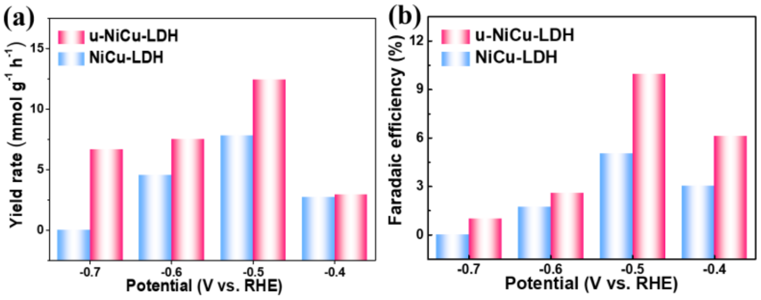

Figure S7. (a) Urea yield rates and (b) Faradaic efficiencies of u-NiCu-LDH and NiCu-LDH under different potentials.

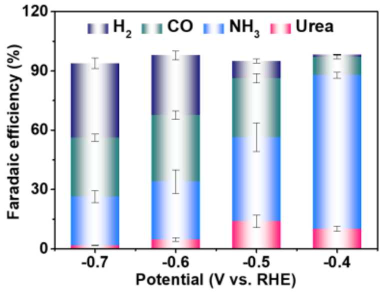

Figure S8. The product distributions during urea synthesis at different potentials on the u-NiCu-LDH/CNF composite.

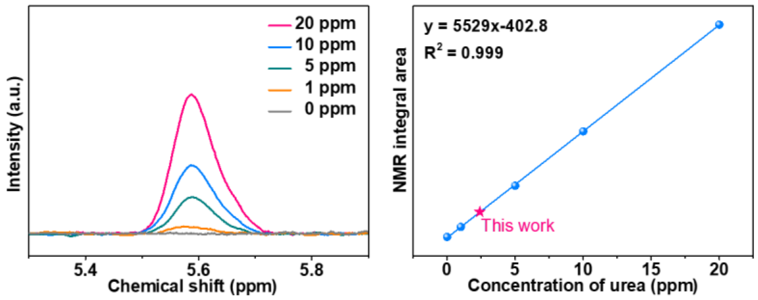

Figure S9. (a) ^1H NMR spectra of urea solutions with various concentrations. (b) The calibration curve for urea solutions with known concentrations.

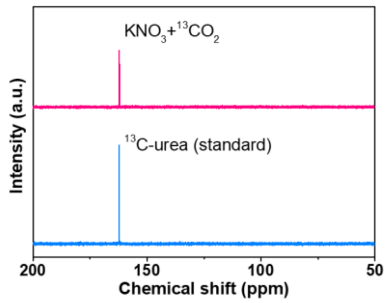

Figure S10. ^{13}C NMR spectra of $^{13}CO_2$-saturated 0.1 M KNO_3 electrolyte at -0.5 V vs. RHE for 2 h and standard ^{13}C-labelled urea.

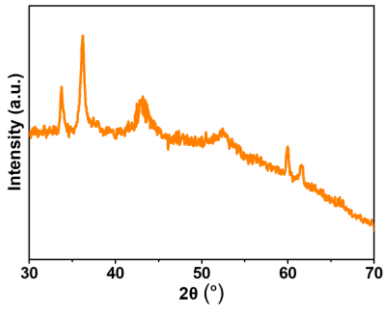

Figure S11. The XRD pattern of the u-NiCu-LDH/CNF composite after long-term stability test.

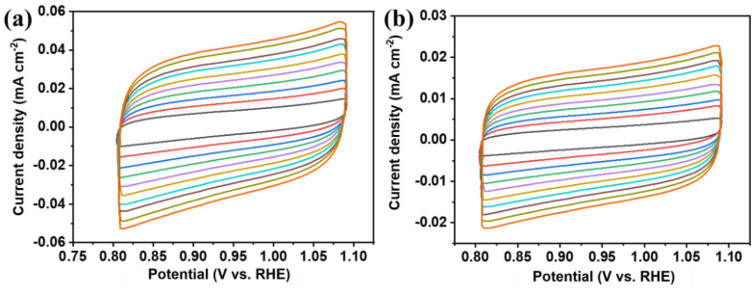

Figure S12. Cyclic voltammograms of (a) u-NiCu-LDH/CNF and (b) NiCu-LDH/CNF composites at different scan rates from 10 to 100 mV s^{-1}.

Table S1. ICP-OES analysis results of the u-NiCu-LDH/CNF and NiCu-LDH/CNF composites.

Catalyst	Cu (wt%)	Ni (wt%)
u-NiCu-LDH/CNF	6.1	11.2
NiCu-LDH/CNF	6.3	11.7

Table S2. Summarization of some recently reported electrocatalysts for urea production.

Catalysts	N source	Urea yield rate	Faradaic efficiency	Stability	Ref.
AuPd nanoalloy	NO_3^-	3.40 mmol h^{-1} g^{-1}	15.60%	20 h	[4]
AuCu nanofibers	NO_2^-	64.83 mmol h^{-1} g^{-1}	24.70%	10 h	[5]
Pd_1Cu_1-TiO_2-400	N_2	3.36 mmol h^{-1} g^{-1}	8.92%	12 h	[6]
Cu@Zn nanowires	NO_3^-	7.29 μmol h^{-1} cm^{-2}	9.28%		[7]
Fe(a)@C-Fe_3O_4/CNTs	NO_3^-	22.35 mmol h^{-1} g^{-1}	16.50%	10 h	[8]
Bi-$BiVO_4$	N_2	5.91 mmol h^{-1} g^{-1}	12.55%	10 h	[9]
$Ni_3(BO_3)_2$-150	N_2	9.70 mmol h^{-1} g^{-1}	20.36%		[10]
u-NiCu-LDH/CNF	NO_3^-	19.43 mmol g^{-1} h^{-1}	13.95%	>20 h	This work

Reference

[1] G. Kresse, J. Furthmuller, *Phys. Rev. B* **1996**, 54, 11169.

[2] G. Kresse, J. Hafner, *Phys. Rev. B* **1993**, 48, 13115.

[3] G. Kresse, J. Furthmüller, *Comput. Mater. Sci.* **1996**, 6, 15.

[4] H. Wang, Y. Jiang, S. Li, F. Gou, X. Liu, Y. Jiang, W. Luo, W. Shen, R. He, M. Li, *Appl. Catal. B-environ.* **2022**, 318, 121819.

[5] S. Liu, S. Yin, Z. Wang, Y. Xu, X. Li, L. Wang, H. Wang, *Cell Rep. Phys. Sci.* **2022**, 3, 100869.

[6] C. Chen, X. Zhu, X. Wen, Y. Zhou, L. Zhou, H. Li, L. Tao, Q. Li, S. Du, T. Liu, D. Yan, C. Xie, Y. Zou, Y. Wang, R. Chen, J. Huo, Y. Li, J. Cheng, H. Su, X. Zhao, W. Cheng, Q. Liu, H. Lin, J. Luo, J. Chen, M. Dong, K. Cheng, C. Li, S. Wang, *Nat. Chem.* **2020**, 12, 717.

[7] N. Meng, X. Ma, C. Wang, Y. Wang, R. Yang, J. Shao, Y. Huang, Y. Xu, B. Zhang, Y. Yu, *ACS Nano* **2022**, 16, 9095.

[8] J. Geng, S. Ji, M. Jin, C. Zhang, M. Xu, G. Wang, C. Liang, H. Zhang, *Angew. Chem. Int. Ed.* **2023**, 62, e202210958.

[9] M. Yuan, J. Chen, Y. Bai, Z. Liu, J. Zhang, T. Zhao, Q. Wang, S. Li, H. He, G. Zhang, *Angew. Chem. Int. Ed.* **2021**, 60, 10910.

[10] M. Yuan, J. Chen, Y. Xu, R. Liu, T. Zhao, J. Zhang, Z. Ren, Z. Liu, C. Streb, H. He, C. Yang, S. Zhang, G. Zhang, *Energy Environ. Sci.* **2021**, 14, 6605.
